# Impact Factors on Migration of Molybdenum(VI) from the Simulated Trade Effluent Using Membrane Chemical Reactor Combined with Carrier in the Mixed Renewal Solutions

**DOI:** 10.3390/toxics10080438

**Published:** 2022-07-31

**Authors:** Liang Pei, Liying Sun

**Affiliations:** 1 Key Laboratory of Water Cycle and Related Land Surface Processes, Institute of Geographic Sciences and Natural Resources Research, Chinese Academy of Sciences, Beijing 100101, China; peiliang@ms.xjb.ac.cn; 2 Xinjiang Institute of Ecology and Geography, Chinese Academy of Sciences, Urumqi 830011, China; 3 University of Chinese Academy of Sciences, Beijing 100049, China

**Keywords:** flat membrane, N,N′-di(1-methyl-pentyl)acetamide, molybdenum, membrane chemical reactor, renewal solution

## Abstract

Molybdenum is harmful and useful. The efficiency of molybdenum trade effluent treatment is low and it is difficult to extract and recycle. To solve this problem, a novel membrane chemical reactor with mixed organic-water solvent(MCR-OW) had been used for the investigation of impact factors on the migration characteristics of Mo(VI) in the simulated trade effluent. The novel MCR-OW contains three parts, such as feeding pool, reacting pool and renewal pool. Flat membrane of polyvinylidene fluoride(PVDF) membrane was used in the reacting pool, the mixed solutions of diesel and NaOH with N, N′-di(1-methyl-pentyl)-acetamide(N-503) as the carrier in the renewal pool and the simulated trade effluent with Mo(VI) as feeding solution. The influencing factors of pH and the ion strength in the feeding solutions, the volume ratio of diesel to NaOH solution and N-503 concentration in the renewal solutions were investigated for the testing of the migration efficiency of Mo(VI). It was found that the migration efficiency of Mo(VI) could reach 94.3% in 225 min, when the concentration of carrier(N-503) was 0.21 mol/L, the volume ratio of diesel to NaOH in the renewal pool was 4:3, pH in the feeding pool was 3.80 and the initial concentration of Mo(VI) was 2.50 × 10^−4^ mol/L. Moreover, the stability and feasibility of MCR-OW were discussed according to Mo(VI) retention on the membrane and the reuse of the membrane.

## 1. Introduction

China has the largest reserves of molybdenum in the world, concentrated in central and north of China with the maximum percentage of porphyry molybdenum deposits (77.5%), following by the porphyry–skarns, skarns and veins [1,2,3]. Molybdenum is widely used in aerospace, metallurgy, steel, electronics, military, petrochemical and agricultural fields, especially playing significant roles in iron and steel industry for alloys [4].

China has so many metal mines, many of which are rich in various toxic and harmful metals. At present, for some political reasons, we need to excavate these minerals. However, these metal mines will also produce a large amount of trade effluent that is difficult to treat. There is a molybdenum mine in a province in northern China. Molybdenum trade effluent is large, which has a certain impact on human and natural environment. The discharge of molybdenum trade effluent is not up to standard. We detected a certain amount of molybdenum in the plant roots around the mine. If this problem is not solved, human consumption of these plants will cause damage to human viscera. The removal effect of traditional effluent treatment methods was not good. This study attempted to explore the method of extracting molybdenum and provide theoretical support for the treatment of molybdenum containing trade effluent in the future. The dramatic economic development has resulted in the rapid consumption of raw materials and increased the related environmental hazards greatly in China [3]. With the large consumptions of molybdenum resources in different industries, the molybdenum contaminations have attracted large attentions in recent years [5,6,7]. Varying degrees of molybdenum pollution was reported in many areas of China and leading to the excessive amounts of molybdenum(VI) in rivers and reservoirs [8,9,10,11]. The transformation of molybdenum contaminants in water can cause toxicological risks to aquatic wildlife and threaten human health [6,11,12]. It was reported that the normal human requirement of molybdenum is up to 300 μg per day with the maximum tolerable does as 2000 μg per day from food, whilst the higher intakes are detrimental to human health [13]. Therefore, the effective method is not only to remove the molybdenum ion from the trade effluent, but also to enrich the molybdenum for the reusing, where understanding the migration of molybdenum ions is the basis.

Great efforts have been made in recent years to develop technologies for the removal and separation of molybdenum in trade effluent, mainly included the chemical precipitation [14,15,16], sub-exchange resin method [17,18], adsorption method [19,20,21], membrane separation [22,23,24], constructed wetland [23,25,26], solvent extraction [27,28,29], biologicalmethods [30]. However, these technologies are still in the development stage, and there are many challenges to be addressed, such as instability, long running time, low removal rate, high energy consumption, secondary pollution and high cost etc., due to the high complexity of the migration processes of the molybdenum ions under different conditions [26].

In the past decades, the migration, removal and reuse of heavy metals from waste water by liquid membranes(LMs), like supported liquid membrane(SLM), emulsion liquid membrane(ELM) and bulk liquid membrane(BLM), coupled with variations of carriers(N-503, P507 and TBP) were recognized to has broad application prospects, due to its advantages of high speed and short time in operation, high enrichment ratio, less reagent consuming and low cost [31,32,33,34,35,36].Among these technologies, SLM is widely used, particularly due to its convenience in operation and low costs in surfactant.

However, the unstable membrane with the less absorption capability of carrier in the SLM technologies remains to over come. It was found that the migration efficiency of heavy metal was higher when organic solvent was introduced into the SLM system [36,37,38]. Still, the stability of the membrane should be addressed. Thus, we designed the renewable membrane chemical reactor with mixed organic-water solvent(MCR-OW) on the basis of SLM for the purposes of both improving the efficiency of molybdenum(VI) ions and the stability of the membrane. N-503 was used as carrier and the diesel was used as the organic solvent. The influencing factors on the migration of Mo(VI) and the stability of the novel MCR-OW were discussed with the expectation of breakthrough industrial applications in future. So there are still some gaps in ore trade effluent treatment technology, which we will just fill the gap with the MCR-OW in the field of ore trade effluent treatment.

To summarize, the purpose of this study is to solve the problems of molybdenum pollution and difficult extraction and recovery in industrial wastewater, and to develop more efficient, more environmental friendly and more stable molybdenum extraction and recovery technology. At the same time, it provides a theoretical and scientific basis for future industrial applications

## 2. Materials and Methods

### 2.1. The Design of MCR-OW and Reactionmechanisms

**Figure 1:**
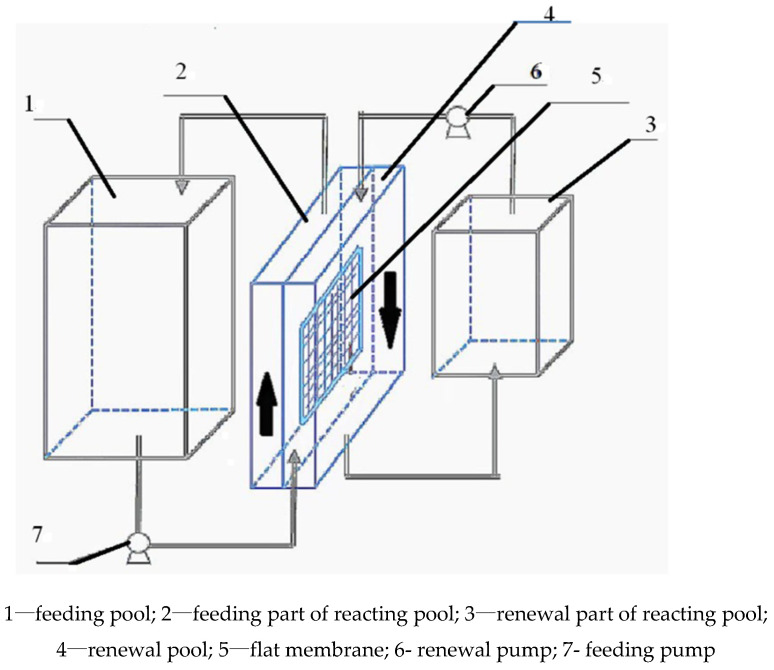
The structure of the membrane chemical reactor with mixed organic-water solvent (MCR-OW).

The framework of MCR-OW was shown in Figure 1. Basically, MCR-OW contained three pools, namely feeding pool (400 mL), reacting pool (300 mL) and enrichment pool (200 mL), connected with the feeding pump and renewal pump. The reacting pool was separated into feeding part and renewal part by the flat membrane, with the effective volume of each part of the reacting pool as 150 mL. Mo(VI) and buffer solution (HAC-NaAc) in the feeding pool was poured into the feeding part of the reacting pool by the feeding pump. The certain volume ratio of the mixed organic-water solution, included membrane solution(diesel) with carrier N-503 and stripping solution(NaOH), in the renewal pool was placed into the renewal part of the reacting pool by the renewal pump for the extraction of Mo(VI) and renewal of the membrane simultaneously, as to improve the stability and increase the extraction rate of Mo(VI) at the same time.

The co-removing involves various (equilibrium) reactions, as Equations (1)–(3):

(1) $$[N-503{]}_{m}+H_{f}^{+}+{Cl}_{f}^{-}={\left([N-503]H^{+}{Cl}^{-}\right)}_{m}$$

(2) $$6{\left([N-503]H^{+}{Cl}^{-}\right)}_{m}+{\left({Mo}_{7}O_{24}^{6-}\right)}_{f}=[{\left([N-503]{H)}_{6}{Mo}_{7}O_{24}\right]}_{m}+6{Cl}_{f}^{-}$$

(3) $${\left[([N-503]H{)}_{6}{Mo}_{7}O_{24}\right]}_{m}+6{\left({OH}^{-}\right)}_{r}\;=6[N-503{]}_{m}+{\left({Mo}_{7}O_{24}^{6-}\right)}_{r}+6{\left(H_{2}O\right)}_{r}$$

Accordingly, the mechanisms included: (a) the diffusion of Mo(VI) from the feeding part into the interface between membrane and the feeding solution; (b) the extraction of Mo(VI) from the feeding solution with carrier N-503 in diesel, resulted in the metal-complex [([N-503]H)_6_Mo_7_O_24_], which expressed as chemical Equations (1) and (2); (c) the diffusion of metal-complex [([N-503]H)_6_Mo_7_O_24_] through the membrane from the interface between membrane and feeding solution of the feeding part to the interface between membrane and renewal solution of the renewal part in the reacting pool; (d) the decomplexation of [([N-503]H)_6_Mo_7_O_24_] into N-503 and Mo(VI) in the interface between membrane and renewal solution, with the NaOH as stripping agent of Mo(VI) and diesel as the solvent of N-503 in the renewal part of the reacting pool, as Equation (3); (e) the enrichment of Mo(VI) in the renewal pool; (f) the re-input of the N-503 by the renewal pump from the renewal pool into the reacting pool, which could react with the feeding solution to increase the stability of the system, due to the diffusion of the N-503 through membrane.

### 2.2. Materials and Reagent

Flat membrane of porous polyvinylidene fluoride(PVDF) membrane was used in our new design, with pore size as 0.24 μm., thickness as 75 μm, tortuosity as 1.67, andporosity as 70~80% (Shanghai Yadong nuclear grade resin Co., Ltd. Shanghai, China). N, N′-di(1-methyl-pentyl)-acetamide(N-503) was used as the carrier in this work, with the density as 0.865, purity as 96% (Shanghai laiyashi Chemical Co., Ltd. Shanghai, China). The HCl and Mo(VI) solutions were mixed as feeding solution to simulate the trade effluent containing Mo(VI). The HAc-NaAc buffer solution was used for the pH adjustment (2.0–6.0) of the feeding solution and the mixed solution of NaCl and KNO_3_ were used for the regulation of the ion strengthin feeding solution to simulate the waste industrial water. NaOH is selected as the stripping solution and the self-made diesel is used as the organic solvent. The mixed solutions ofdiesel with N-503 and NaOH solution were used as the renewal solution. All the reagents (except diesel) were of analytical grade.

### 2.3. Test Method

Digital Acidity Ion Meter(pHS-3C, Shanghai Kangyi Instrument Co., Ltd. Shanghai, China) is used to determine the pH of the solution. The concentration of Mo(VI) was determined by spectrophotometry with 4-(2-pyridine-azo) resorcinol(PAR) as chromogenic agent and the absorbance was measured at 530 nm. The migration efficiency(*Re*) and separation coefficient are calculated as the logarithm value of the ratio of Mo(VI) concentration (*C_t_*) to the initial Mo(VI) concentration(*C_0_*) in the feeding pool, shown in Equation (4) and the migration rate(*Rr*) is calculated as Equation (5).

(4) $$Me=-\ln\left(\frac{C_{t}}{C_{0}}\right)$$

(5) $$Mr=\frac{C_{0}-C_{t}}{C_{0}}\times 100$$

### 2.4. Experimental Procedure

All experiments were accomplished at 20 ± 5 °C in the MCR-OW. The effective area of the device is 20 cm^2^. The flow rates of two pumps are all 11.3 mL/min. Before the experiment, the PVDF membrane was firstly immersed into the diesel solvent with N-503 for an hour and then dried naturally and fixed into the reacting pool. The prepared feeding solution and renewal solution were poured into feeding pool and renewal pool separately. Then the experiments were initiated formally with the starting of both feeding pump and renewal pump. Samples were taken from the feeding pool and renewal pool for the tests of the Mo(VI) concentration, at 30, 60, 100, 165, 225 min, respectively.

## 3. Results and Discussion

### 3.1. Effects of pH and Ion Strength in the Feeding Pool

**Figure 2:**
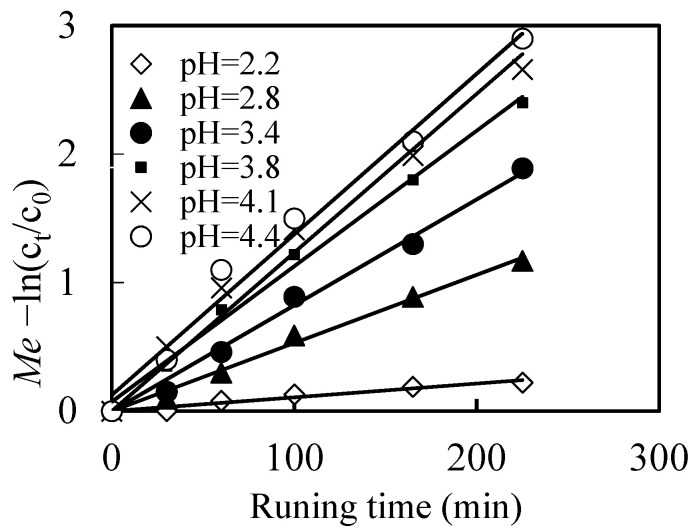
Effects of pH of the feeding solution on the migration efficiency (*Me*) of Mo(VI).

Basically, the concentration difference between feed part and renewal part in the reacting pool is the driving power of mass removing process, which should be impacted by the pH of the feeding solution [39]. To investigate the effects of pH of the feeding solution on the migration efficiency(*Me*) of Mo(VI) in our work, the initial experimental conditions were that: ratio of diesel toNaOHin the renewal pool ast1:1, the concentration of NaOH solution at 0.30 mol/L, the concentration of carrier at 0.21 mol/L in the mixed solutions of the renewal pool. The initial concentration of Mo(VI) was adjusted to 2.50 ×10^−4^ mol/L in the feeding pool. As shown in Figure 2, *Me* increased when pH of the feeding solution increased from 2.2 to 4.4, and increased with the increase of the running time as well.

According to Equations (1) and (2), when H^+^ increased, it is benefit for both the formations of ([N-503]H^+^Cl^-^) and [([N-503]H)_6_Mo_7_O_24_]. Also, pH is the driving power of the mass diffusion of the complex [([N-503]H)_6_Mo_7_O_24_], which is theoretically increased the migration efficiency of Mo(VI) when pH decreased. However, in our experiment, we found that molybdenum existed in the form of MoO_2_^2-^when pH decreased, which hinder the reactions of Equation (2) and lower the migration efficiency of Mo(VI). As a result, the lower the pH is, the more inefficiency the *Me* is. We chose pH of 3.80 as the optimum pH condition of the feed part during the following experiments.

**Figure 3:**
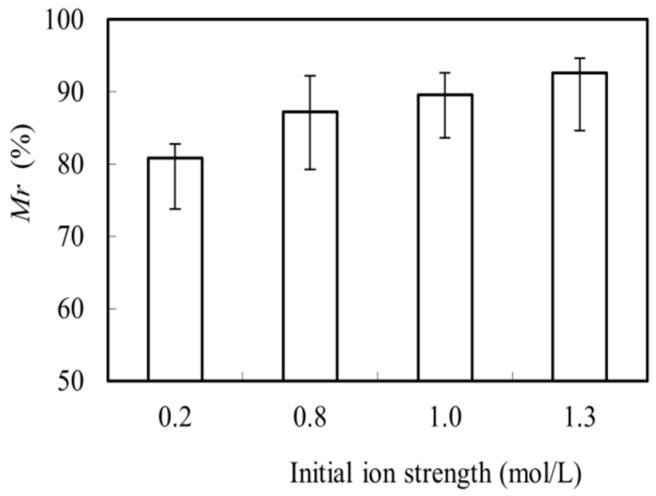
Effects of the initial ion strength in the feeding pool on the migration rate (*Mr*) of Mo(VI).

In the trade effluent, there always existed various ions, which may affect the migration of Mo(VI). Thus the NaCl and KNO_3_ were used to simulate and regulation ion strength of the simulated trade effluent. The effects of the initial ion strength in the feeding pool on the migration rate (*Mr*) of Mo(VI) was thus examined. As shown in Figure 3, the *Mr* of Mo(VI) increased when the initial ion strength changed from 0.2 mol/L to 1.3 mol/L, kept higher than 80%. Ionic strength has little impact on the MCR-OW system, which is more suitable for industrial application and lays a foundation for future industrial application [40].

### 3.2. Effects of the Volume Ratio and Carrier Concentration in the Renewal Pool

**Figure 4:**
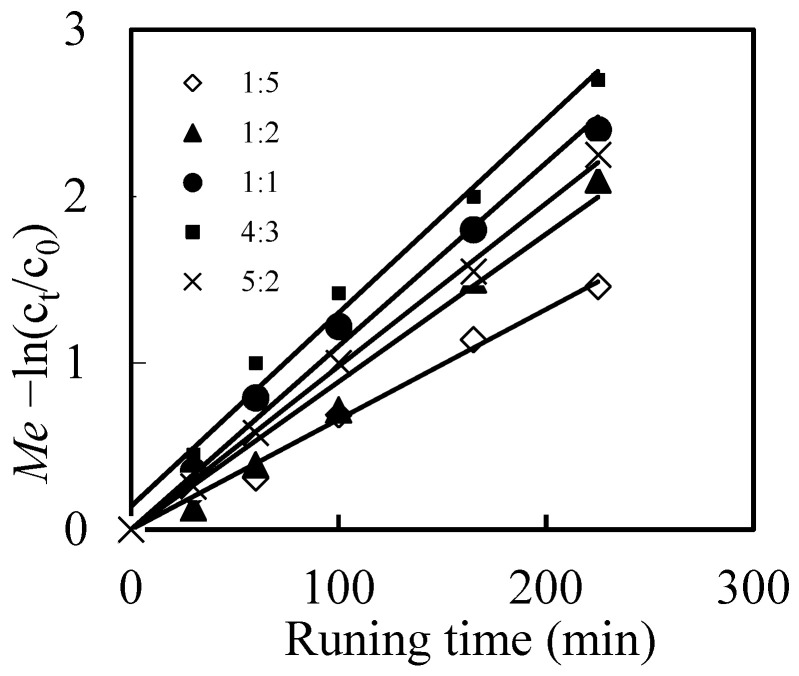
Effects of the volume ratio of diesel to NaOH on the migration efficiency (*Me*) of Mo(VI).

As we described above, the mixed solutions in the renewal pool was a certain volume ratio of diesel with N-503 to NaOH. To investigate the volume ratio effects of the mixed solutions, the ratio of diesel to NaOH was adjusted as 1:5, 1:2, 1:1, 4:3, 5:2, and the results were shown in Figure 4. The migration efficiency(*Me*) of Mo(VI) firstly increased with the volume ratio of diesel to NaOH, when it lower than 4:3. And then *Me* of Mo(VI) decreased sharply when the volume ration of diesel to NaOH reached 5:2.

In our work, the diesel with N-503 was selected as membrane solution, which could recover the stability of the membrane and benefit the formation of the complex compounds, by increasing the chances of the recycling of the N-503. Simultaneously, the NaOH was selected as stripping solution for the migration of Mo(VI), the increase of which not only increased the stripping rate for the decomplexation of [([N-503]H)_6_Mo_7_O_24_], but also increased the concentration differences of the H^+^ concentration in the feeding part and renewal part of the reacting pool, to increase the diffusion of both [([N-503]H)_6_Mo_7_O_24_] and the recycled N-503. When ratio of diesel to NaOH increased, the complexation rate increased and the stripping rate and diffusion rate decreased, the balance of these reactions resulted in the maximum migration efficiency of Mo(VI) when volume ratio of diesel to NaOH was 4:3.

**Figure 5:**
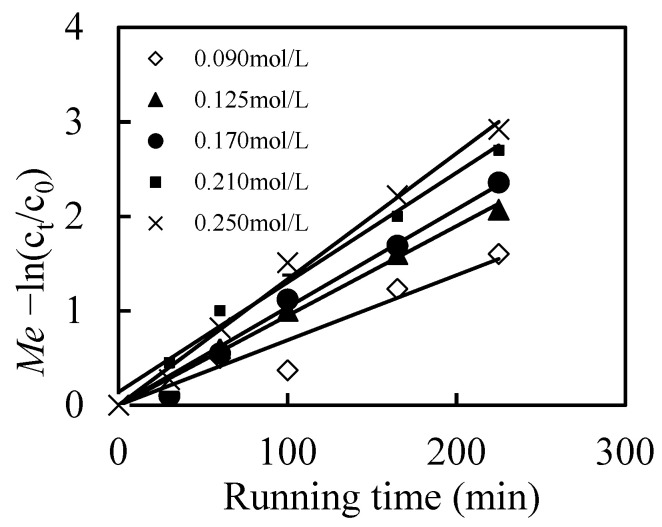
Effects of N-503 concentration on the migration efficiency (*Me*) of Mo(VI).

Moreover, N-503 played important roles for the migration efficiency of the Mo(VI). Based on Equations (1) and (2), the higher N-503 is, the higher chances of the formation of complexation [([N-503]H)_6_Mo_7_O_24_] are, which increased the migration efficiency (*Me*) of Mo(VI) (Figure 5). It was noticed that the differences on *Me* of Mo(VI) was much when the N-503 concentration changed from 0.21 mol/L to 0.25 mol/L. Considering the removal efficiency and solvent cost, 0.21 mol/L was selected as the optimum carrier concentration.

### 3.3. Effects of Mo(VI) Retention on the Reuse of the Membrane

**Figure 6:**
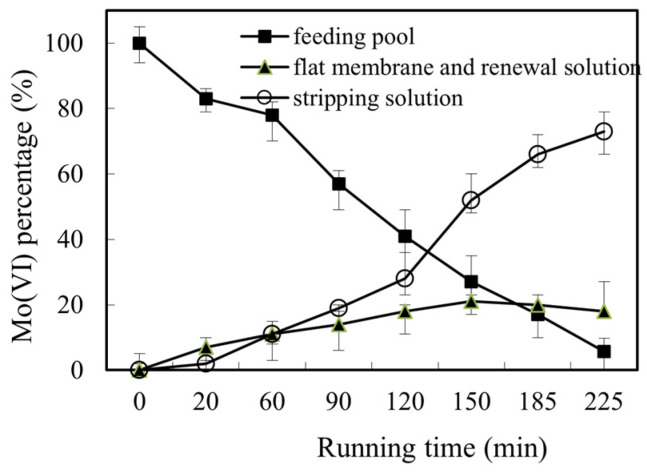
The retention of Mo(VI).

In the previous investigations of the removal of heavy metal using SLM, the retention of the heavy metal ion on the membrane was observed and concerned [32,33]. In this work, the Mo(VI) ion was also concerned. According to the concentration of Mo(VI) in both feed pool and renewal pool, the concentration of Mo(VI) on the membrane can be calculated. As shown in Figure 6, the retention of Mo(VI) on the membrane increased with the running time. However, the increase rate decreased with the running time, which resulted in the the stable concentration of Mo(VI) percentage on the membrane when running time was longer than 150 min. This is mainly due to the decrease of the decomplexation rate of [([N-503]H)_6_Mo_7_O_24_] in the interface between membrane and renewal solution with increase of Mo(VI) in the renewal part of the reacting pool when running time increased [41]. With the increasing of the running time, the balance reached and the retention of Mo(VI) no longer increased, at the approximately 18% of the Mo(VI) on the membrane of the reacting pool.

**Figure 7:**
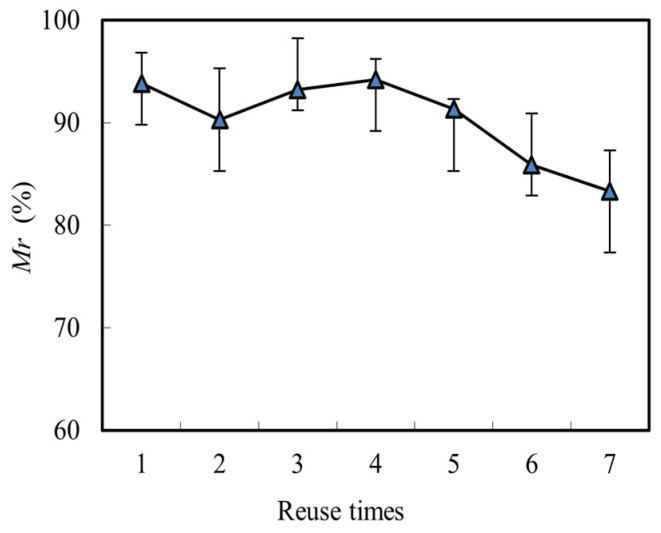
The effects of reuse time on the migration rate (*Mr*) of Mo(VI).

However, the retention of Mo(VI) on the membrane did not have significant negative effects on the renewal of themembrane. As shown in Figure 7, the migration rate(*Mr*) kept higher than 80% when the experiment repeated 7 times, which verified the stability and feasibility of the MCR-OW. The stability of the membrane and the migration rate of Mo(VI) was also enhanced by the separation of the reacting pool with the feeding and renewal pools in the novel design. This is precisely the advantage of our new designed MCR-OW, which avoid the falls off of the carrier that may be caused by the stirrer in the traditional SLM method. This part of the research has great reference significance for our future industrial application of the MCR-OW.

## 4. Conclusions

The influencing factors on the migration characteristics of Mo(VI) from the simulated trade effluent was examined using our new designed membrane chemical reactor with mixed organic-water solvent(MCR-OW) in this work. The results showed that MCR-OW was able to improving the efficiency of Mo(VI) migration from the simulated trade effluent, using the mixed diesel and NaOH(stripping solution) with the carrier of N-503 as renewal solution. The migration efficiency(*Me*) of Mo(VI) increased with the increase of the pH in the feeding pool, the concentration of the carrier(N-503) in the renewal pool and the running time and the concentration. The migration rate(*Mr*) kept higher than 80% when the initial ion strength of other ions increased from 0.2–1.3 mol/L. *Me* of Mo(VI) firstly increased with the volume ratio of diesel to NaOH (≤4:3) and then decreased sharply. Considering on both the removal efficiency and the solvent costs, the optimum parameters of the MCR-OW were chosen as follows: the concentration of carrier(N-503) at 0.21 mol/L, the volume ratio of diesel to NaOH in the renew alpool at 4:3, pH in the feeding pool at 3.80, the running time at 225 min. Under the optimum conditions, the migration rate(*Mr*) could reach 94.3% when the initial Mo(VI) concentration was 2.50 × 10^−4^ mol/L. Although there are detainment or retention of Mo(VI) on the membrane during the operation, it did not hinder the reuse of the membrane with the *Mr* of Mo(VI) higher than 80% even though the membrane was used seven times. However, the retention of the membrane should be further tackled in the future for the application of the MCR-OW in larger scale.

Although the research is still in the experimental stage at present, as long as the state pays more attention to the research and development of new technologies for the treatment of trade effluent and environmental protection policies support the extraction and recycling of special substances, this technology will be widely used. In the future, we will do some pilot research with enterprises to explore its practical application effect. In the future, the development of material technology will greatly reduce the price of membrane, and the cost of this technology will be greatly reduced. At that time, there will be no problem to widely promote and apply it.

## Data Availability

The datasets used and/or analyzed during the current study are avail-able from the corresponding author on reasonable request.
